# Cell maps on the human genome

**DOI:** 10.1186/s13039-019-0426-4

**Published:** 2019-03-20

**Authors:** Christopher Cherniak, Raul Rodriguez-Esteban

**Affiliations:** Committee for Philosophy and the Sciences, Department of Philosophy, University of Maryland, College Park, MD 20742 USA

**Keywords:** Genome, Somatotopic map, Homunculus, “Cellunculus”, Organelle-specific gene, Connection optimization

## Abstract

**Background:**

We have previously described evidence for a statistically significant, global, supra-chromosomal representation of the human body that appears to stretch over the entire genome.

**Results:**

Here, we extend the genome mapping model, zooming down to the typical individual animal cell. Its cellular organization appears to be significantly mapped onto the human genome: Evidence is reported for a “cellunculus” — on the model of a homunculus, on the *H. sapiens* genome.

**Conclusions:**

Basic cell structure turns out to map similarly onto the total genome, mirrored via genes that express in particular cell organelles (e.g., “nuclear membrane”). Similar cell maps may also appear on individual chromosomes that map topologically on the dorsoventral body axis. This seems to constitute some of the basic structural and functional organization of nucleus and chromosome architecture.

**Electronic supplementary material:**

The online version of this article (10.1186/s13039-019-0426-4) contains supplementary material, which is available to authorized users.

## Background

This report proceeds from body maps to cell maps. We converge from macro-scale down to micro-scale: We test a genome mapping model for the individual eukaryotic animal cell. Results are described for significant reflection of cell organization in gene patterns on the human genome.

In plots of mean positions on the genome’s central–peripheral axis of genes expressing in each of 10 major cell organelles (from “nucleus” to “plasma membrane”) vs corresponding positions of the organelles themselves within the typical animal cell, the cell-genome correlation is significant (as strong as *p* < 0.004).

As for the body maps reported earlier [1], each of the individual organelle-gene distribution trends by itself is nonsignificant; but the “trend of trends” progression of the set of these slopes together seems significant.

We also report evidence suggesting cell maps localize on individual dorsoventral [DV] chromosomes—i.e., chromosomes that map the dorsoventral axis of the body. This DV cell map is significantly stronger than cell maps on anteroposterior [AP] chromosomes.

Previously, for body maps on individual chromosomes, we had found a “division of labor” for individual chromosomes: Half of the chromosomes appear to represent the DV body axis, the other half the AP body axis (See Table 2, in [2]). Here, we also find cell mappings are more significant on DV chromosomes than on AP ones. In addition, when our earlier division of labor findings for the body map DV axis on DV chromosomes are combined with similar results for cell maps on DV chromosomes, a functional rationale emerges for observed clustering of DV chromosomes in the core of the sperm cell nucleus.

The underlying framework of the research program here is “genome as palimpsest” — that is, a maps-within-maps model. The human genome appears to have overlapping layers of various somatic mappings intercalated at different scales. This report focusses on maps of cell microstructure, along with maps of the human body outlined earlier elsewhere [1].

As discussed previously, one functional explanation for these maps might be that they help minimize message-passing costs within the genome (See [3] for a similar account of connection-optimization in the brain).

## Methods

**Fig. 1:**
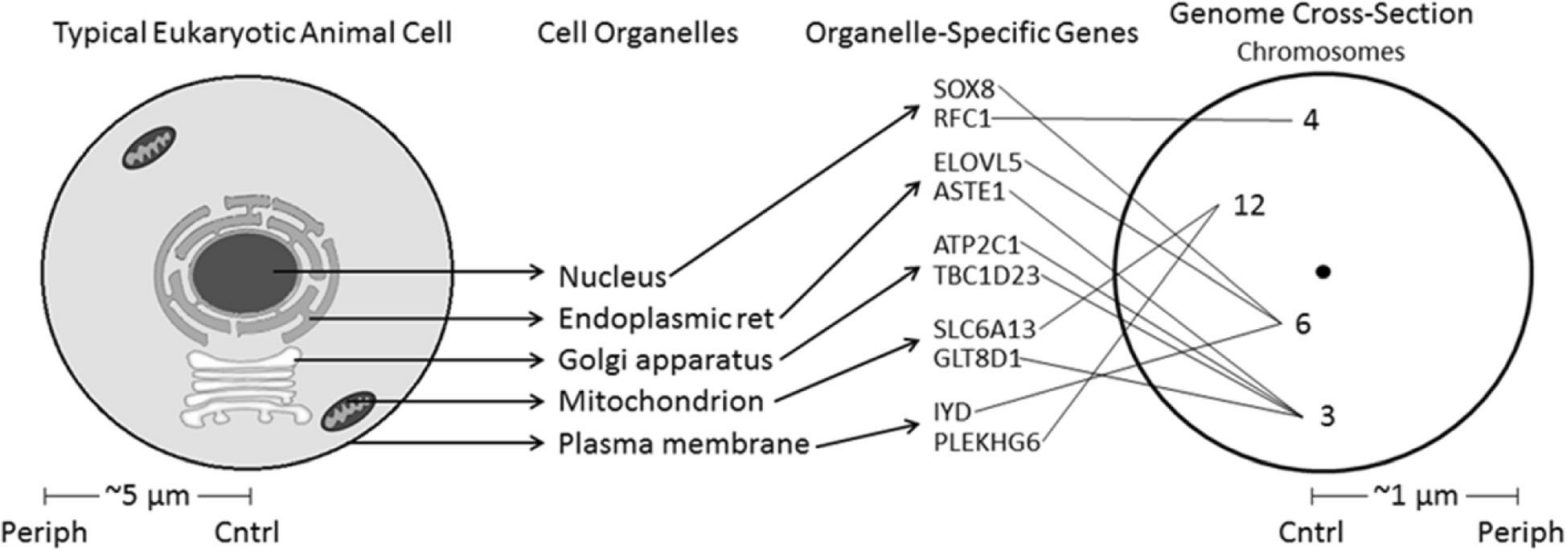
A first approximation: Mapping the typical eukaryotic animal cell onto the human genome, on the central-peripheral axis. Five cell organelles of the ten examined are illustrated. For each organelle, two of the genes that express uniquely in that organelle are shown (derived from [5]). Each gene is then traced to its chromosome. Approximate chromosome sites in the sperm cell nucleus are indicated (based on Additional file 1: Table S2, in [1]). So, organelle → genes → chromosomes → nucleus locations

Figure 1 diagrams the scheme here for evaluating a cell-genome mapping hypothesis. We start with a cell anatomy model based on the familiar observation of approximate radial organization of the typical eukaryotic animal cell plan.

For instance, on Google, under, e.g., “cell diagram”, etc., are hundreds of images (some copying from others), with comparatively few disagreements on the basic radial map of cell organelle positions, from center (nucleus) to periphery (plasma membrane). A familiar illustration of this groundplan is [4].

Because of its extensive, consistent, and recent curation, the “Human Protein Atlas” [5, 6] is used here. The cell schematic then is [7]. For explanation of cell-anatomical positions of each organelle, see [8]. (See also “Locate” subcellular localization database [9]).

Cell organelles were excluded from this analysis that were not topologically compact on their radial axis (e.g., centrosome vs plasma membrane). Ten organelles then remain. In center-to-periphery order: Nucleus, Nucleolus Fibrillar Center, Nucleolus, Nuclear Speckle, Nuclear Body, Nuclear Membrane; Endoplasmic Reticulum, Golgi Apparatus, Mitochondrion, Plasma Membrane.

We compiled Additional file 1: Table S1, a datafile containing our full Protein Atlas genecount datatable. A mean total of 37 distinct genes are expressed in each organelle included. The human Y chromosome has the smallest total gene count, and so does not appear in the present analyses.

It should be observed that, unlike the TiSGeD tissue gene database [10] used for our earlier study of body maps on chromosomes, the Protein Atlas database here does not include information on how preferentially a gene expresses in a given target (here, a cell organelle). Therefore, as a first approximation, we next include only genes that each express uniquely in a single type of organelle.

One question is whether this select geneset would suffice to map cell component genes onto the whole genome, as in our report [1] on tissue gene body maps. Another issue is whether the genecounts of the Protein Atlas database would suffice to filter for the most selectively-expressed genes.—For instance, for genes that each uniquely express in only one cell component. Or, would such a restriction reduce genesets so much that too many empty cells arise in the resulting main (Additional file 1: Table S2)?

To attempt in this way to boost resolution and sharpen focus of a cell map on the genome, genes maximally specific for *H. sapiens* cell organelles were identified that are listed as expressing for only *one* organelle (e.g., for “nucleolus”). For each such cell component, there are a mean 10 such uniquely expressing genes per chromosome. None of the organelles here in fact occur with empty (0) selective gene counts for 1/3 or more of the 23 chromosomes.

Also accessible is Additional file 1: Table S2, with this select Protein Atlas genecount dataset. The original full Protein Atlas data Additional file 1: Table S1 includes 8558 distinct genes. The maximally select data Additional file 1: Table S2 consists of 2325 genes that each express uniquely in only a single organelle, i.e., 27% of the original full total geneset.

**Fig. 2:**
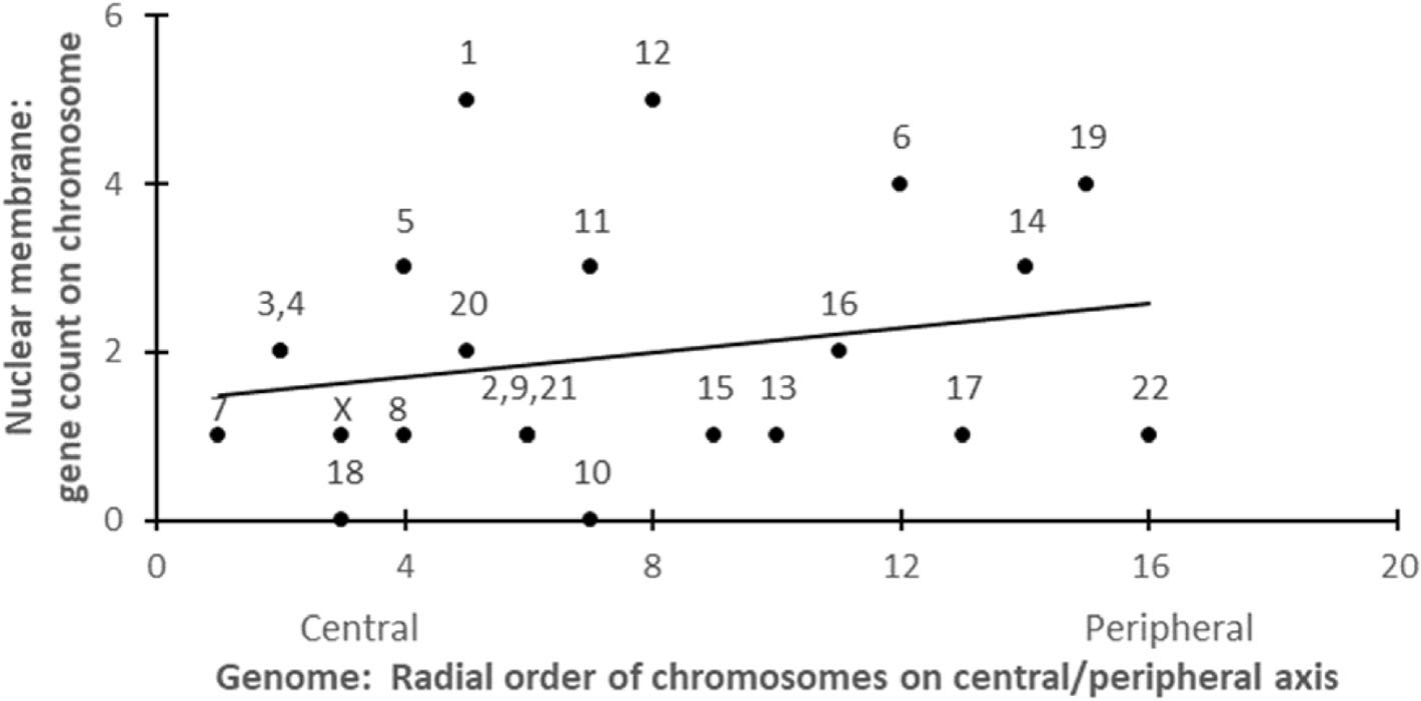
Typical example of distribution of organelle-specific genes on 23 chromosomes in the human sperm cell genome: here, genes each uniquely expressing in “nuclear membrane” of cell. (See Additional file 1: Table S2.) The positive distribution trend is not strong (*r*^*2*^ = 0.05); however, when all 10 such sets of organelle-specific genes are fitted together, a statistically strong trend emerges (cf. Figure 3 below). Each datapoint is labelled with its chromosome number. (Chromosomes 2, 9, and 21 share same genome site on central-peripheral axis, and same organelle-specific gene counts; similarly for chromosomes 3 and 4)

For locating organelle genes in the total genome, chromosome positions can be identified in the sperm cell genome via Additional file 1: Table S2 in [1]. (See Fig. 2 gene distribution example below.)

## Results

### Cell maps on the genome

Three successively stronger replications of the cell-genome mapping result are reported here: A simple linear model for the trendlines appears to suffice. For a conservative estimate of statistical significance, a 2-tail (symmetrical, bidirectional) distribution model was employed.

**Fig. 3:**
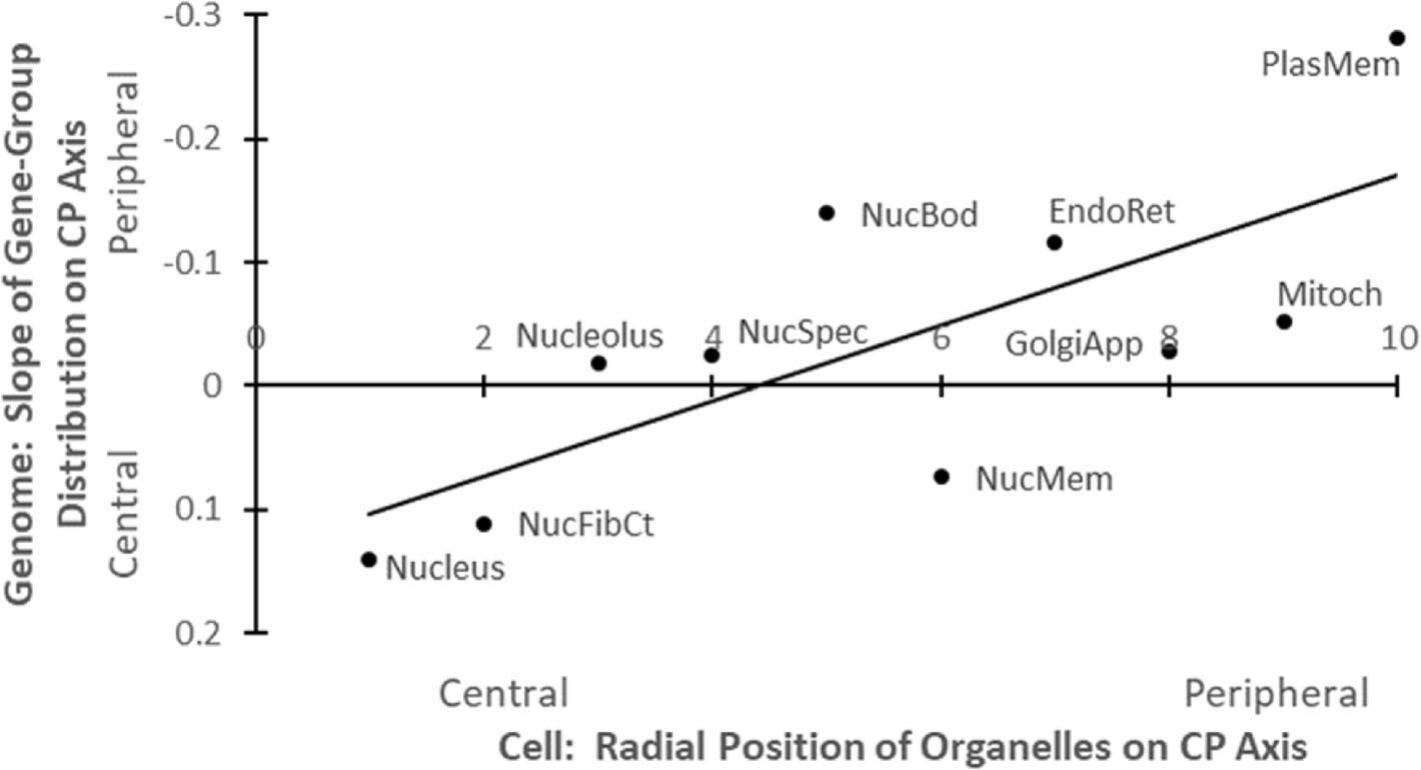
Isomorphism of cell microanatomy and largescale human genome structure: Components positioned more centrally in a cell tend to have their genes correspondingly concentrated on chromosomes sited more toward the center of genome. — For the maximally selective subset of the Human Protein Atlas (Additional file 1: Table S2), where each gene expresses uniquely in only one organelle. Each datapoint is labelled with its organelle-name (see Table 1)

1. For the original full Human Protein Atlas (Additional file 1: Table S1), as opposed to the select Human Protein Atlas, including all genes expressing in the 10 organelles, the cell map on the genome already shows a significant pattern (*r*^*2*^ = 0.494, *p* < 0.024, 2 tail).
2. For the select Human Protein Atlas (Additional file 1: Table S2), and Table 1, in the Fig. 3 plot below of the 10 organelles, a similar cell-genome correlation is significant and stronger (*r*^*2*^ = 0.540, *p* < 0.015, 2 tail).
3. With datapoints each weighted by their own magnitude of effect *r*^*2*^ (as in [1]): In a plot of the 10 organelles, the cell-genome correlation further increases in significance (to: *r*^*2*^ = 0.677, *p* < 0.004, 2 tail).

**Table 1:** Cell organelles: Their Central-Peripheral [CP] positions in cell, and the gradient of their genes’ distribution in the genome

Central		CellAnat	(Slope)	*r* ^*2*^	Select GeneCt
		Cell CP Order	GeneCt Gradient		
Nucleus	**Nucleus**	1	0.1397	0.0027	463
Nucleolus Fib Ctr	**NucFibCtr**	2	0.1112	0.095	41
Nucleolus	**Nucleolus**	3	−0.0184	0.0003	178
Nuclear Speckle	**NucSpec**	4	−0.0253	0.0003	221
Nuclear Body	**NucBod**	5	−0.1395	0.0792	83
Nuclear Membrane	**NucMem**	6	0.0730	0.0488	45
Endoplasmic Ret	**EndoRet**	7	−0.1155	0.0105	223
Golgi Apparatus	**GolgiAp**	8	−0.0280	0.0003	253
Mitochondrion	**Mitoch**	9	−0.0520	0.0005	574
Plasma Membrane	**PlasMem**	10	−0.2816	0.0449	244
Peripheral		Means	−0.0336	0.0283	232.5
		Total			2325

(For explanation of cell-anatomical positions of organelles, see [7, 8])

(Abbreviations of organelle names in Fig. 3 are listed in boldface.) Each gene expresses uniquely in one organelle-type

Earlier, we have reported comparable correlation patterns for mapping the human body onto the human genome (cf. Figures four, five, six in [1]). Again, each individual organelle trend by itself is nonsignificant; but the “trend of trends” progression of the set of these slopes pooled together is significant.

In Fig. 1, the isomorphism of the central/peripheral cell cross-section with the dorsoventral genome cross-section, but not with the orthogonal anteroposterior (head / tail) genome cross-section (cf. Figure one, in [1]), is evident. The correlations of Fig. 3 hold for organelle and gene positions on the central / peripheral axis of the typical cell and genome; in contrast, for the orthogonal head / tail genome axis, the pattern is not significant (*r*^*2*^ = 0.163, *p* < 0.248, 2 tail).

### Cell maps on chromosomes

Progressing down to a finer scale, we now examine cell maps on individual chromosomes. (See additional summary Additional file 1: Table S3.)

A next question is, Are there cell maps for individual chromosomes resembling those we reported for the entire genome? Once more, each of the individual trends by itself is nonsignificant; but a “trend of trends” cumulative progression of the set of these slopes together approaches significance. Aggregating the 22 autosomal correlations yields some mapping results.

As we have reported [2], as well as body maps on the complete genome, body maps on individual chromosomes are strongly significant. For instance, our earlier report described corresponding mappings of the human body onto the human genome [1], and our subsequent paper reports significant similar body mappings onto individual chromosomes [2]. It should be noted that, if similar significant cell mappings appear also on chromosomes, that suggests such cell maps are widespread throughout many types of eukaryotic cells. — In particular, not only on the haploid spermcell genome, but also on diploid genomes.

As mentioned earlier, gene expression databases for cell organelles do not seem to include measures of preferential strength of gene expression in a given organelle type, while we found gene expression databases for the earlier body map analyses that did include such relative magnitude of expression. So, cell map detection should not be as sensitive as body map detection. Therefore, a prediction to test is whether gene databases for cell organelles that do include such measures of gene expression selectivity strength in fact will reveal more of cell map structure on chromosomes.

Nonetheless, as mentioned above for cell maps on the complete genome, the set of chromosome cell map correlations is similarly stronger for the DV than the AP axis of the genome. Next, comparing *r*^*2*^ magnitudes of cell maps on DV vs AP chromosomes: See earlier chromosome “division of labor,” Table two, in [2]. In this way, cell maps on individual DV chromosomes also seem stronger than those on AP chromosomes. This constitutes further independent converging support of the earlier DV vs AP chromosome division of labor for body maps in [2]. (Of the 11 AP chromosomes, Chrs 21 and 11 had the two weakest body map *r*^*2*^ values; in this respect, they are the most marginal members of the AP group).

**Fig. 4:**
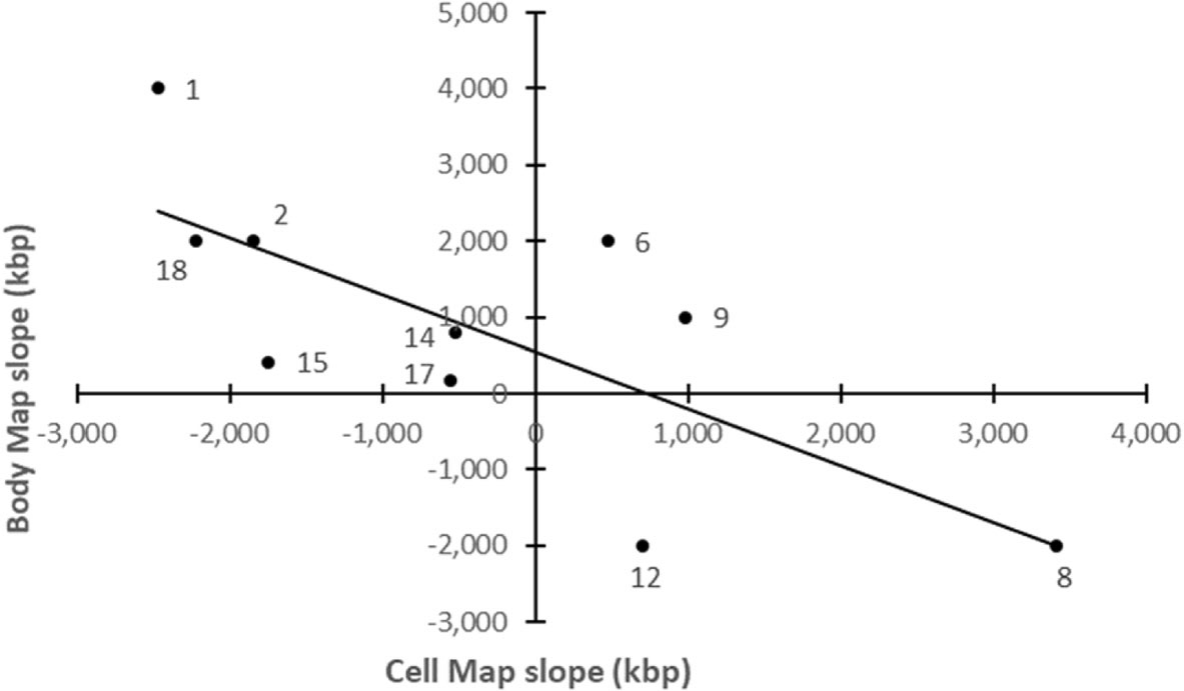
Body map - cell map relationship on DV chromosomes. For each DV chromosome, its body map slope and cell map slope tend to be inversely related (*r*^2^ = 0.543, *p* < 0.015). That is, the more positive the body map gradient, the more negative the cell map gradient, and vice versa. (Each datapoint is labelled with its DV chromosome number.) In contrast, AP chromosomes show no significant body map - cell map relationship. Nor do *r*^*2*^ values of body maps and cell maps show a significant relationship

Instead, for mean slope values of cell maps on DV vs AP chromosomes: The DV chromosome set has a mean 25% greater (steeper) slope than the AP chromosome set (*p* < 0.087, 2 tail). In addition, for mean *r*^2^ values of body maps vs cell maps on DV chromosomes: On DV chromosomes, cell maps have a mean 9% stronger *r*^*2*^ value than corresponding body maps (*p* < 0.056, 2 tail). See also Fig. 4 below. In these ways, cell maps appear stronger than body maps. So, some mapping of cell anatomy onto AP instead of DV chromosomes is detectable.

**Fig. 5:**
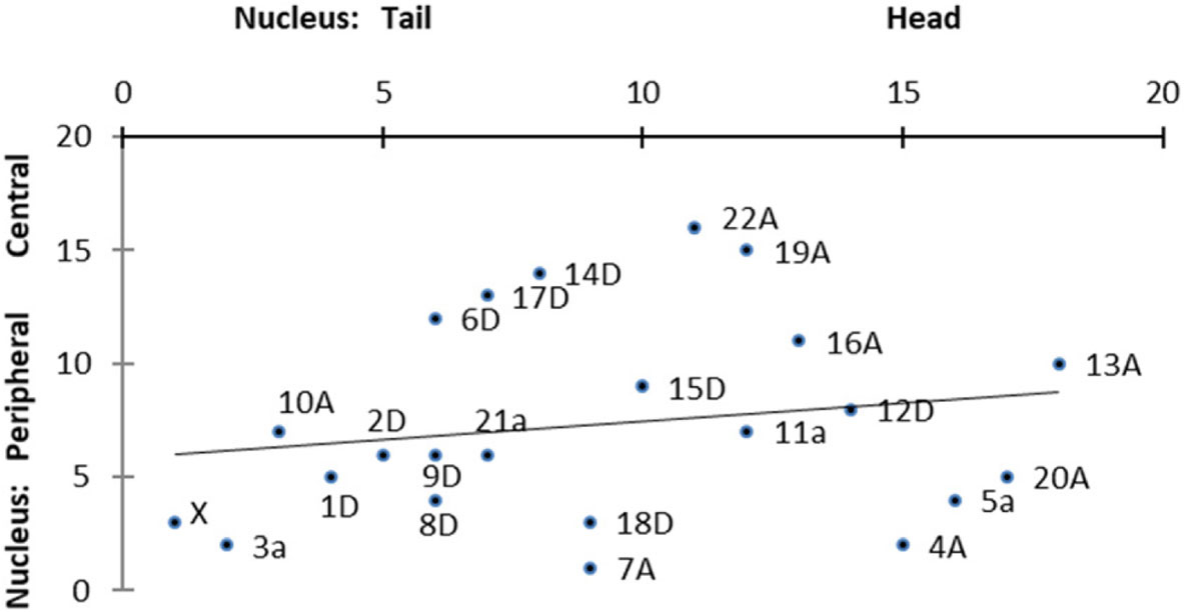
Partial map of centroids of chromosome sites in *H. sapiens* sperm cell nucleus (updated). **A**, chromosomes with AP body map; **D**, chromosomes with DV body map (Chrs 3 and 5 are marginally AP). Each chromosome group appears to have a topologically distinct meta-territory in the nucleus: Anteroposterior chromosomes tend to occupy an anterior outer border region (with exception of Chrs 11 and 21), which surrounds an inner core that dorsoventral chromosomes occupy. (Of the 11 AP chromosomes, 11a and 21a have the two lowest AP *r*^*2*^ values; in this way, they are the weakest (most marginal) members of the AP group.) Each axis gives position-order of chromosomes. (Nucleus map is constructed from Tables S1 and S2, in [1]; based on Figures two and four of [12]). Best fit line for all 23 chromosome positions is included

In addition, for further localization of cell maps: In the sperm cell nucleus, the DV chromosome cluster is positioned significantly rearward of the AP cluster (*p* < 0.011, 2 tail); so, on the head-tail axis, the cell map chromosomes group in the posterior of the nucleus (see Fig. 5).

## Conclusion

Global genome structure and function: In the human sperm cell nucleus, the concentration of cell maps on DV, not AP chromosomes, suggests an explanation for the significant central cluster of DV chromosomes in the genome (See Fig. 4, in [2]).

A functional rationale can be discerned for grouping cell map chromosomes in such a core, surrounded by a shell of AP chromosomes — as opposed to vice versa (instead positioning DV chromosomes in the shell, or mixing DV and AP sites). Such separation would tend to minimize distances between cell organelle genes, thereby reducing message-traffic costs among cell genes. This improves the match with a typical cell, which has message-propagation distances that are orders of magnitude smaller than such distances in the entire body of an organism.

Another clustering rationale along similar lines: As a germ cell, the sperm cell has a haploid nucleus. Adult somatic cells are diploid, and do not show the DV-core/AP-shell configuration. (E.g., see [11]). One interpretation for this difference would be that intracellular message-passing peaks early in the developmental trajectory.

In this way, these cell map findings for the sperm cell also provide independent convergent support, and a functional explanation, for similar earlier body map results regarding the global “core / shell” layout of DV vs AP chromosomes. (See Fig. 5).

How, if at all, do these cartographic phenomena relate to the rest of genetic physiology? Is so extensive a structure as a genomic map merely functionless ornament upon the genome’s terra incognita? As mentioned earlier, a design rationale for this mapping might be that such maps help economize costs of interconnections in the genetic system. With whole-genome surveys of recent decades, examining such global organization of the human genome landscape seems a natural next step. To start, as convergent confirmation, we have positive preliminary results that similarly map *C. elegans* “worm brain” anatomy onto its 6 chromosomes.

## Additional file

Additional file 1: **Table S1.** GeneCts per chromosome and cellular location. Gene entries include all subcellular targets. **Table S2.** GeneCts per chromosome and cellular location. Only for genes each with a single subcellular target. **Table S3.** Mean position of each cell organelle-specific gene set on its chromosome. (PDF 134 kb)

## Abbreviations

AP: Anteroposterior
Chr: Chromosome
CP: Central-peripheral
DV: Dorsoventral
GeneCt: Genecount
