# Supplementary material for: Cell maps on the human genome
Source: Mol Cytogenet. 2019 Mar 20;12:14. doi: 10.1186/s13039-019-0426-4 (PMC6425595; doi:10.1186/s13039-019-0426-4)
Supplement: Supplementary file 1 — Table S1. GeneCts per chromosome and cellular location. Gene entries include all subcellular targets. Table S2. GeneCts per chromosome and cellular location. Only for genes each with a single subcellular target. Table S3. Mean position of each cell organelle-specific gene set on its chromosome. (PDF 134 kb) [file 13039_2019_426_MOESM1_ESM.pdf]

FULL Cherniak & Rodriguez-Esteban. Cell maps on the human genome.

Table S1. GeneCts per chromosome and cellular location. (Gene entries include **all** subcellular targets.) Website: Human Protein Atlas available on www.proteinatlas.org
Compiled from: **full** Human Protein Atlas v16.1 [Thul P, et al, 2017. A subcellular map of the human proteome. Science 356(6340)].

Cell anatomy	Chromosome	**1**	**2**	**3**	**4**	**5**	**6**	**7**	**8**	**9**	**10**	**11**	**12**	**13**	**14**	**15**	**16**	**17**	**18**	**19**	**20**	**21**	**22**	**X**	**MeanCts**	**Totals**
	Cell Organelle																									
Center	**Nucleus**	173	137	103	73	84	98	87	62	93	85	121	101	28	73	56	81	95	28	160	43	19	43	78	83.52	1921
	**Nucleolus fib ctr**	20	21	11	11	13	10	9	8	7	15	18	7	5	8	7	13	13	5	22	11	3	5	10	10.96	252
	**Nucleolus**	127	104	59	51	61	53	49	51	52	58	73	57	22	46	36	53	69	15	86	37	16	26	65	55.04	1266
Ordered by position	**Nuclear speckles**	58	23	25	11	20	31	21	14	13	13	24	20	4	14	12	25	27	8	33	14	3	12	19	19.3	444
on radial axis	**Nuclear bodies**	43	35	30	20	22	22	15	20	18	24	25	22	10	16	17	20	32	5	37	12	5	17	15	20.96	482
	**Nuclear membrane**	23	27	7	15	11	13	15	8	8	9	19	14	5	11	7	10	17	5	24	8	5	5	6	11.83	272
	**Endoplasmic ret**	36	31	26	16	24	20	18	21	13	22	28	20	8	14	13	21	21	7	26	17	1	9	18	18.7	430
	**Golgi apparatus**	103	50	55	37	46	43	46	33	39	42	65	49	15	27	34	39	55	19	69	25	6	17	45	41.7	959
	**Mitochondrion**	106	75	70	45	48	47	50	32	42	57	73	55	14	41	27	42	56	18	65	17	10	38	38	46.35	1066
Periphery	**Plasma membrane**	148	94	84	52	82	72	71	46	53	59	87	88	30	45	45	60	77	27	92	38	15	33	68	63.74	1466
	[10 of 27 organelles total]																									
	Mean Gene Counts	83.7	59.7	47	33.1	41.1	40.9	38.1	29.5	33.8	38.4	53.3	43.3	14.1	29.5	25.4	36.4	46.2	13.7	61.4	22.2	8.3	20.5	36.2	**37.21**	
	Totals	837	597	470	331	411	409	381	295	338	384	533	433	141	295	254	364	462	137	614	222	83	205	362		**8558**

Range of gene counts for an organelle: 1 - 173 genes (i.e., for each gene expressed in one or more organelles).
Mean count of **all** genes for each organelle: 37.2 genes per chromosome. (Vs total all genes for each chromosome: 372.1)

SELECT Cherniak & Rodriguez-Esteban. Cell maps on the human genome.

Table S2. GeneCts per chromosome and cellular location. (Only for genes each with a **single** subcellular target.) Website: Human Protein Atlas available on www.proteinatlas.org
For genome cell map: Compiled from Human Protein Atlas v 16.1 [Thul P, et al, 2017. A subcellular map of the human proteome. Science. 356(6340)].

Cell anatomy		Chromosome	**1**	**2**	**3**	**4**	**5**	**6**	**7**	**8**	**9**	**10**	**11**	**12**	**13**	**14**	**15**	**16**	**17**	**18**	**19**	**20**	**21**	**22**	**X**	**Mean Cts**	**Totals**
		Cell Organelle																									
Center	1	**Nucleus**	52	30	25	12	18	29	31	15	25	16	23	22	6	15	10	18	26	6	45	13	5	7	14	20.13	463
	2	**Nucleolus fib ctr**	5	2	2	2	1	2	1	1	1	0	0	0	2	1	0	5	2	1	6	2	2	2	1	1.783	41
	3	**Nucleolus**	18	13	8	10	8	4	11	9	7	6	7	8	3	3	1	10	15	3	18	4	4	3	5	7.739	178
Radial axis	4	**Nuclear speckles**	32	12	15	7	14	15	6	7	6	3	13	14	3	5	4	11	12	3	17	8	3	3	8	9.601	221
	5	**Nuclear bodies**	5	3	3	5	9	3	6	4	6	5	3	2	3	3	3	5	1	0	6	4	0	0	4	3.609	83
	6	**Nuclear membrane**	5	1	2	2	3	4	1	1	1	0	3	5	1	3	1	2	1	0	4	2	1	1	1	1.957	45
	7	**Endoplasmic ret**	25	16	14	8	10	11	12	8	7	11	12	10	5	8	4	12	11	3	10	12	0	7	7	9.696	223
	8	**Golgi apparatus**	29	14	17	8	12	10	14	3	14	10	18	11	2	7	8	8	14	6	25	8	1	5	9	11	253
	9	**Mitochondrion**	57	41	28	30	28	21	33	15	25	37	40	21	6	25	13	23	32	10	36	9	6	15	23	24.957	574
Periphery	10	**Plasma membrane**	25	17	18	7	14	8	11	11	12	7	19	17	3	5	6	10	12	5	15	7	2	4	9	10.609	244
		Mean Gene Counts	25.3	14.9	13.2	9.1	11.7	10.7	12.6	7.4	10.4	9.5	13.8	11	3.4	7.5	5	10.4	12.6	3.7	18.2	6.9	2.4	4.7	8.1	**10.11**	
		Totals	253	149	132	91	117	107	126	74	104	95	138	110	34	75	50	104	126	37	182	69	24	47	81	**101**	**2325**

[10 of 27 organelles total. Filtered by: compact distribution in cell. And less than 1/3 of chromosomes have 0 genes uniquely expressing in each organelle.]

Range of gene counts for an organelle: 1 - 57 maximally selective genes (i.e., each gene expressed in only one organelle).
Mean maximally selective gene count for each organelle: 10.1 genes per chromosome. (Vs **all** genes for each organelle included: 37.2)

CHROMO Cherniak & Rodriguez-Esteban. Cell maps on the human genome.

Table S3. Mean position of each cell organelle-specific gene set on its chromosome (in base units, from p-arm origin).
For chromosome cell maps: Compiled from Human Protein Atlas v 16.1 [Thul P, et al, 2017. A subcellular map of the human proteome. Science. 356(6340)].
Each gene position on a chromosome is derived from the Human Protein Atlas entry itself (mean of start and end sites of each gene). Website: Human Protein Atlas available at www.proteinatlas.org

	Chromosome		1	2	3	4	5	6	7	8	9	10	11	12	13	14	15	16	17	18	19	20	21	22	X	
	Cell	Organelle																								
Center	1	**Nucleus**	1.08E+08	9.70E+07	1.08E+08	6.68E+07	1.09E+08	6.87E+07	9.31E+07	1.00E+08	9.35E+07	8.51E+07	8.30E+07	6.95E+07	5.55E+07	5.81E+07	6.87E+07	4.28E+07	4.44E+07	3.59E+07	3.69E+07	4.06E+07	4.28E+07	3.33E+07	7.81E+07	**Nucleus**
	2	**NucFibCtr**	8.04E+07	1.50E+08	1.05E+08	7.76E+07	5.28E+07	3.70E+07	1.06E+08	1.44E+08	3.78E+07				6.95E+07	5.78E+07		6.40E+07	2.58E+07	3.55E+07	4.00E+07	1.05E+07	3.29E+07	3.72E+07	1.04E+08	**NucFibCtr**
	3	**Nucleolus**	9.34E+07	1.18E+08	8.17E+07	9.32E+07	1.08E+08	3.07E+07	9.01E+07	8.05E+07	7.27E+07	7.85E+07	5.79E+07	5.95E+07	5.95E+07	6.32E+07	4.18E+07	4.40E+07	4.93E+07	2.88E+07	3.59E+07	2.70E+07	3.83E+07	3.25E+07	8.13E+07	**Nucleolus**
	4	**NucSpec**	1.07E+08	1.73E+08	9.78E+07	9.94E+07	1.17E+08	5.47E+07	1.05E+08	8.23E+07	1.02E+08	6.03E+07	6.65E+07	6.74E+07	4.83E+07	6.61E+07	6.36E+07	3.23E+07	4.52E+07	2.31E+07	3.97E+07	3.93E+07	3.80E+07	3.95E+07	7.02E+07	**NucSpec**
	5	**NucBod**	1.14E+08	9.87E+07	2.72E+07	6.25E+07	1.03E+08	7.98E+07	6.18E+07	4.90E+07	1.15E+08	7.08E+07	4.95E+07	2.93E+07	6.81E+07	6.22E+07	5.78E+07	5.76E+07	4.01E+07		3.34E+07	2.26E+07			6.79E+07	**NucBod**
	6	**NucMem**	2.14E+08	2.19E+08	5.14E+07	1.25E+08	6.52E+07	3.29E+07	8.57E+05	1.33E+08	9.66E+07		5.92E+07	6.58E+07	2.62E+07	8.76E+07	7.56E+07	8.11E+07	4.02E+07		2.26E+07	1.68E+07	4.44E+07	3.88E+07	3.46E+07	**NucMem**
	7	**EndoRet**	1.23E+08	1.33E+08	9.86E+07	6.91E+07	1.04E+08	6.72E+07	9.14E+07	5.49E+07	9.49E+07	5.57E+07	6.08E+07	5.37E+07	5.34E+07	5.49E+07	7.40E+07	4.39E+07	4.58E+07	4.32E+07	2.05E+07	4.25E+07		3.41E+07	8.99E+07	**EndoRet**
	8	**GolgiApp**	1.13E+08	1.36E+08	1.03E+08	9.78E+07	1.24E+08	5.49E+07	8.80E+07	7.92E+07	8.57E+07	5.47E+07	5.91E+07	5.65E+07	4.91E+07	5.58E+07	6.21E+07	5.47E+07	4.74E+07	5.46E+07	2.53E+07	4.13E+07	4.55E+07	3.60E+07	7.64E+07	**GolgiApp**
	9	**Mitoch**	1.10E+08	1.22E+08	1.13E+08	8.01E+07	1.00E+08	8.19E+07	8.39E+07	9.33E+07	8.31E+07	8.03E+07	7.44E+07	7.75E+07	3.78E+07	6.82E+07	6.37E+07	3.53E+07	4.58E+07	3.89E+07	2.39E+07	2.40E+07	3.64E+07	3.40E+07	6.83E+07	**Mitoch**
Periphery	10	**PlasMem**	1.33E+08	1.44E+08	1.03E+08	7.49E+07	9.56E+07	5.97E+07	6.67E+07	1.04E+08	8.01E+07	9.99E+07	6.58E+07	3.08E+07	7.54E+07	6.96E+07	5.77E+07	3.01E+07	3.28E+07	4.42E+07	3.72E+07	3.61E+07	3.96E+07	4.28E+07	9.30E+07	**PlasMem**

[10 of 27 organelles total. Filtered by: compact distribution in cell. And less than 1/3 of chromos have 0 genes uniquely expressing in each organelle.]

Range of gene counts for an organelle: 1 - 57 maximally selective genes (that is, for each gene expressed in only **one** organelle).
Mean maximally selective gene count for each organelle: 10.1 genes per chromosome. (Vs all genes for each organelle included: 37.2)

(In order to compact the original ChromoCellMap table S3 so that it can be easily printed out, the cellformat of the numerical values has been converted to exponential representation. (E.g., in the above rendering of the table, the mean position-value on Ch 1 of the "nucleus"-specific genes is 1.08E+08 bp ; however, in the original Excel file, the explicit format of this value is still retained as 108166622.3 bp .)
